# Amyloid, iron and cerebrovasculopathy in a new mouse model combining hemochromatosis mutations and Alzheimer’s amyloidosis and the etiological and diagnostic implications for dementia subtypes

**DOI:** 10.1002/alz.085295

**Published:** 2025-01-03

**Authors:** Adrienne (Lz) Milward, Ritambhara Aryal, Elvis Acquah, Daniel Johnstone

**Affiliations:** ^1^ University of Wollongong, Wollongong, NSW Australia; ^2^ University of Newcastle, Callaghan, NSW Australia; ^3^ University of Utah, Salt Lake City, UT USA

## Abstract

**Background:**

UK Biobank data show mutations related to the iron disorder hemochromatosis can approximately double the risk of dementia, in particular clinically diagnosed vascular dementia. Insights into the etiology of this dementia may be provided by cerebrovasculopathy in our new “Aβ+Iron^dys^” mouse model, which combines hemochromatosis‐related mutations and amyloidosis, with increases in soluble Aβ species and plaques. This was created by crossing an established APP/PS1 model of β‐amyloidosis with our reported *Hfe* ^‐/‐^x*Tfr2* ^mut^ model of hemochromatosis‐related mutations exhibiting brain iron dyshomeostasis (Heidari Mol. Psych. 2016).

**Method:**

Deposition of amyloid and other Aβ species was visualized by Congo red, thioflavins and Aβ immunolabeling. Iron was visualized by diaminobenzidine (DAB)‐enhanced Perls’ or transcardially perfused DAB+Turnbull’s staining.

**Result:**

Compared to regular APP/PS1 mice, Aβ+Iron^dys^ mice have extensive cerebrovasculopathy, sometimes severe, at 6 months and older. This is typically accompanied by iron deposition in vessel walls, sometimes co‐localizing with amyloid (Congo Red birefringence). Cerebrovasculopathy includes enlarged perivascular spaces, tortuosity, wall thickening, lumenal stenosis, microaneurysm, anastomosis and other changes in vessel profiles and diameter, wall rupture and microhemorrhage. Intense parenchymal staining for iron adjoining cortical vessels may arise from ruptured vessels.

**Conclusion:**

Co‐deposition of iron and Aβ within blood vessels is exacerbated by hemochromatosis‐related mutations and can cause severe cerebrovasculopathy and amyloid angiopathy. This could lead to neuronal hypoperfusion and dysfunction throughout the brain in early disease stages and ultimately to neurodegeneraion. This has potential implications for understanding the etiology of dementia related to hemochromatosis mutations and for the clinical diagnosis of dementia subtypes.

